# Predicting progression from MCI to dementia using cortical disarray measurement from diffusion MRI

**DOI:** 10.1002/alz.70310

**Published:** 2025-05-26

**Authors:** Mario Torso, Pegah Khosropanah, Steven A. Chance, Gerard R. Ridgway

**Affiliations:** ^1^ Oxford Brain Diagnostics Ltd Oxford UK

**Keywords:** Alzheimer's disease, cortex, cortical diffusivity, diffusion tensor imaging, mild cognitive impairment, minicolumns, prognosis, survival analysis

## Abstract

**BACKGROUND:**

This study evaluates the capability of cortical microstructural measures from diffusion magnetic resonance imaging (MRI) to predict progression from mild cognitive impairment (MCI) to dementia, compared to commonly used macrostructural measures. Identification of high‐risk individuals can support both clinical practice and trials.

**METHODS:**

Structural and diffusion MRI scans of 826 participants from the National Alzheimer's Coordinating Center (NACC) were analyzed to extract macrostructural measures and three minicolumn‐related diffusivity metrics: AngleR, PerpPD^+^, and ParlPD. Kaplan–Meier survival analysis was used to investigate time to progression to dementia, with participants stratified by biomarker metrics.

**RESULTS:**

Cortical diffusivity (PerpPD^+^ in medial–temporal and connected regions) outperformed hippocampal volume, cortical volume, and cortical thickness in Kaplan–Meier survival analysis, predicting faster conversion to dementia.

**DISCUSSION:**

Cortical microstructural measures from diffusion MRI provide powerful biomarkers for predicting progression from MCI to dementia, offering enhanced prognostic capabilities that could support earlier intervention strategies in clinical practice and improve the power of clinical trials.

**Highlights:**

- Cortical minicolumn‐related diffusivity metrics measure neurodegeneration.
- We compare the predictive value of magnetic resonance imaging (MRI) measures for mild cognitive impairment to dementia progression.
- Microstructural cortical disarray outperforms macrostructural markers.
- These results support using diffusion MRI biomarkers to identify and monitor at‐risk patients.

## 1 INTRODUCTION

Mild cognitive impairment (MCI) is an intermediate stage between normal cognitive aging and dementia characterized by measurable cognitive deficits that do not significantly impair daily functioning. MCI is a heterogeneous condition—not all MCI cases progress to dementia—identifying those at higher risk is crucial for early intervention and treatment planning.^1^ With the approval of amyloid clearance therapies able to slow disease progression, an early diagnosis and an accurate prediction of MCI progression can enable timely therapeutic interventions.

Considering recent efforts to establish a biological definition of Alzheimer's disease (AD) within the context of the amyloid/tau/neurodegeneration (ATN) framework,^2^ the identification of reliable, cost‐effective, non‐invasive biomarkers capable of detecting early signs of the disease is a crucial goal. The significant progress achieved in the development of fluid biomarkers of amyloid and tau represents a major recent advancement in the field.^3^ However, the identification and validation of novel early markers of “neurodegeneration” require further efforts. Although neurofilament light chain (NfL) in blood and cerebrospinal fluid (CSF) shows sensitivity to neurodegenerative processes, its lack of specificity for different types of neurodegeneration^3^, ^4^ and the absence of spatial localization highlights the need for imaging modalities. Imaging biomarkers, potentially alongside NfL, could enhance detection or confirmation of neurodegeneration, monitoring of disease progression and treatment response (favorable or unfavorable), and aid in distinguishing different neurodegenerative diseases.

Magnetic resonance imaging (MRI) has emerged as an important modality, offering non‐invasive insights into structural and functional brain changes associated with neurodegeneration. Beyond conventional imaging techniques that focus on macrostructural changes, microstructural MRI techniques, such as diffusion tensor imaging (DTI), free‐water imaging, or neurite orientation dispersion and density imaging (NODDI), provide a deeper understanding of the brain's microarchitecture in the early stages of the disease.^5^, ^6^, ^7^, ^8^, ^9^, ^10^ These methods can quantify subtle cortical alterations that are increasingly recognized as early indicators of pathology. It has been shown that AD neuropathological changes at in post mortem associated with earlier cortical microstructural changes detectable through in vivo cortical diffusion imaging.^11^

**RESEARCH‐IN‐CONTEXT**

1. **Systematic review**: Macrostructural imaging metrics, such as hippocampal volume, have been extensively studied and are routinely used to assess neurodegeneration. However, recent advancements in diffusion magnetic resonance imaging have enabled the characterization of cortical microstructure, revealing insights into neurodegenerative processes not captured by volumetric analyses. Despite their potential, biologically informed cortical diffusion measures have not been widely implemented, and their value for dementia prognosis remains underexplored.
2. **Interpretation**: Cortical diffusion measures significantly outperform traditional macrostructural markers, in predicting progression from mild cognitive impairment to dementia. Findings highlight the value of cortical microstructure in detecting subtle neurodegeneration, supporting the use of microstructural metrics for early diagnosis and monitoring.
3. **Future directions**: Future research should integrate microstructural measures with other biomarkers, such as positron emission tomography imaging and blood‐based markers, for enhanced predictive accuracy and/or more fine‐grained stratification. Validation in diverse cohorts and investigation with disease‐modifying treatments could further establish clinical utility.

Although many recent studies have demonstrated the promising potential of cortical diffusivity measures in detecting changes across the aging and dementia continuum, to our knowledge, no study has performed survival analyses comparing microstructural measures to widely used macrostructural measures, such as hippocampal volume.

This study investigates a set of minicolumn‐related metrics previously developed and validated using ex vivo and in vivo data^5^, ^11^, ^12^, ^13^ to detect and monitor neurodegeneration along the clinical continuum. Cortical minicolumns are the basic organizational units of the neocortex, consisting of vertically aligned neurons and their associated synaptic networks, and changes in the columnar architecture in AD contribute to the hallmark symptoms of the disease.^14^, ^15^, ^16^

Previous studies have demonstrated how this set of cortical measures is useful in detecting the presence of AD,^13^ in differential diagnosis,^17^, ^18^, ^19^ and in identifying MCI patients at higher risk of conversion to various forms of dementia.^20^ Furthermore, these measures have proven to be sensitive for monitoring patient response in clinical trials.^21^

This study aims to evaluate the ability of cortical diffusivity measures to detect and monitor neurodegeneration along the clinical continuum; to assess the measures’ capability to predict progression from MCI to dementia; and to use survival analysis to compare the performance of the cortical microstructural measure that best distinguishes MCI converters from non‐converters to the performance of three widely used macrostructural measures of neurodegeneration in AD: hippocampal volume, cortical volume, and cortical thickness.

The integration of microstructural data with other modalities, such as molecular biomarkers and cognitive assessments, may further refine prognostic accuracy, paving the way for personalized medicine approaches.

## 2 METHODS

### 2.1 Participants

The present study comprised 826 participants, ranging from cognitively normal to severe dementia, having clinical structural and DTI scans from the National Alzheimer's Coordinating Center (NACC). Table 1 summarizes characteristics of all the participants included in this study.

**TABLE 1:** Clinical and demographic characteristics.

	CU *n* = 474	MCI NC *n* = 110	MCI C *n* = 86	AD *n* = 156
**Clinical and demographics**				
Age, years	65.4 (9.7)	73.1 (8.1)^*^	75.6 (7.6)^*^	73.3 (8.9)^*^
Sex, female	308 (65)	51 (46.4)^*^	34 (39.5)^*^	72 (46.1)^*^
Education, years	16.4 (4.5)	15.9 (2.5)	15.8 (2.9)	15.5 (7.4)
**Race**				
White	432 (91.1)	104 (94.5)	76 (88.4)	138 (88.5)
Black or African American	33 (7)	5 (4.5)	6 (7)	15 (9.6)
American Indian or Alaska Native	2 (0.4)	0 (0)	2 (2.3)	0 (0)
Asian	6 (1.3)	0 (0.0)	2 (2.3)	1 (0.6)
Other	0 (0)	0 (0)	0 (0)	2 (1.3)
Unknown	1 (0.2)	1 (0.9)	0 (0)	0 (0)
**Ethnicity**				
Non‐Hispanic	457 (96.4)^†^	87 (79.1)	78 (90.7)^†^	140 (89.7)^†^
Hispanic	13 (2.7)^†^	23 (20.9)	6 (7)^†^	16 (10.3)^†^
Unknown	4 (0.8)	0 (0)	2 (2.3)	0 (0)
*APOE* ε4 carrier	158 (34.1) N.A. = 10	29 (27.1)^*^ N.A. = 3	49 (59)^*^, ^†^ N.A. = 3	102 (68.4)^*^, ^†^ N.A. = 7
Follow‐up time, years	7.1 (2.7)	4.7 (2.2)	5.7 (2.8)	–
Interval MRI‐conversion, years	–	–	2.9 (2.2)	–
MMSE	29.4 (0.9)	27.1 (1.8)^*^	26.4 (2.2)^*^, ^†^	21.9 (4.7)^*^, ^†^, ^‡^
CDR global	0	0.5 (0.2)	0.5 (0.2)	0.97 (0.5)
CDR‐SB	0.6 (0.3)	0.9 (0.9)^*^	2 (1.4)^*^	5.4 (3.2)^*^

	CU *n* = 104	MCI NC *n* = 8	MCI C *n* = 15	AD *n* = 39
**CSF biomarkers**				
Aβ_1–42_ (pg/mL)	734.19 (200.1)	610.97 (237.6)	369.91 (126.7)^*^, ^†^	415.89 (206.9)^*^, ^†^
P‐tau_181P_ (pg/mL)	41.41 (16.8)	42.78 (20.1)	69.58 (46.1)	92.08 (46.4)^*^ ^,#^
T‐tau (pg/mL)	305.71 (153.5)	294.17 (162.6)	615.06 (353.3)	725.24 (387.2)

	CU *n* = 474	MCI NC *n* = 110	MCI C *n* = 86	AD *n* = 156
**Macrostructural MRI**				
Cortical Volume Fraction	0.299 (0.02)	0.280 (0.02)	0.273 (0.02)^*^	0.263 (0.03)^*^, ^†^, ^‡^
Cortical Thickness	2.48 (0.09)	2.43 (0.10)	2.38 (0.10)^*^, ^†^	2.33 (0.12)^*^, ^†^, ^‡^
Cortical Thickness Signature	2.57 (0.10)	2.53 (0.12)	2.44 (0.11)^*^, ^†^	2.37 (0.15)^*^, ^†^, ^‡^
Hippocampal Volume Fraction	0.0052 (0.0005)	0.0048 (0.0006)	0.0042 (0.0006)^*^, ^†^	0.0041 (0.0007)^*^, ^†^
Ventricles Volume Fraction	0.018 (0.009)	0.026 (0.010)	0.030 (0.011)^*^, ^†^	0.034 (0.013)^*^, ^†^
White Matter Hypointensities	0.0015 (0.007)	0.0030 (0.003)	0.0036 (0.004)	0.0052 (0.007)^*^, ^†^

*Note*: Continuous factors reported as mean (standard deviation) and categorical factors reported as *n* (%).

Mean value and standard deviation for each measure were reported. Analysis of variance was used for continuous variables and chi‐square tests were used for categorical variables. Marginal means tests were adjusted for multiple comparisons using Bonferroni correction.

Abbreviations: Aβ, amyloid beta; AD, Alzheimer's disease; *APOE*, apolipoprotein E; CDR, Clinical Dementia Rating; CDR‐SB, Clinical Dementia Rating Sum of Boxes; CSF, cerebrospinal fluid; CU, cognitively unimpaired; MCI, mild cognitive impairment; MCI C, mild cognitive impairment converter; MCI NC, mild cognitive impairment non‐converter; MMSE, Mini‐Mental State Examination; MRI, magnetic resonance imaging; N.A ., not available; p‐tau, phosphorylated tau; t‐tau, total tau.

* Significantly different compared to control (Bonferroni‐corrected *P*  <  0.05).

^†^ Significantly different compared to MCI NC (Bonferroni‐corrected *P*  <  0.05).

^‡^ Significantly different compared to MCI C (Bonferroni‐corrected *P*  <  0.05).

The NACC database (https://naccdata.org/) provides data from patients collected in the Alzheimer's Disease Research Centers (ADRCs) funded by the National Institute on Aging. This analysis used data from 15 ADRCs collected between 2009 and 2023.

Inclusion was limited to participants who were cognitively unimpaired (CU) or had a clinical AD‐type presentation (AD, MCI) who had undergone MRI scanning (T1‐weighted structural and DTI) and clinical evaluation. For preliminary analysis, all participants were categorized, using longitudinal follow‐up data, into one of the following subgroups: (1) Cognitively unimpaired (CU = 474): Individuals with normal cognition (global Clinical Dementia Rating [CDR] = 0 and/or neuropsychological testing within normal range) and normal behavior, who remained CU for all available follow‐up visits. All the CU participants included in the study have at least 2 years of follow up. (2) MCI non‐converter (MCI NC, *N* = 110): Individuals with objective cognitive deficits who did not progress to dementia during the available follow‐up periods. Only participants with a minimum follow‐up of 2 years were included. (3) MCI converter (MCI C, *N* = 86): Individuals with objective cognitive deficits at baseline who progressed to dementia at any follow‐up visit after the MRI scan. (4) AD (*N* = 156): Participants with clinical diagnosis of dementia at baseline and amyloid biomarker confirmation of AD pathology when available (amyloid positron emission tomography [PET] or CSF amyloid beta [Aβ]42 or autopsy confirmation).

Note that the survival analysis (described below) includes all 196 MCI participants and does not make use of the MCI C and MCI NC groups as defined above.

To characterize the cohort, the Mini‐Mental State Examination (MMSE), apolipoprotein E (*APOE*) ε4, and CDR global and sum of boxes (CDR‐SB) scores were used.

For a subset of participants, measurements of Aβ42, total tau (t‐tau), and phosphorylated tau (p‐tau) were available, obtained from CSF samples. The CSF biomarkers were analyzed using two assay methodologies: ELISA (enzyme‐linked immunosorbent assay) and Luminex technology.

### 2.2 MRI acquisition and image preprocessing

For each participant, the DTI and 3D T1‐weighted structural images were used. Data was acquired from 3T MRI scanners (Siemens and GE Medical Systems) at multiple centers. For more details about structural and diffusion acquisition protocols, see www.alz.washington.edu. Diffusion scans with slice thickness > 3 mm or b‐value > 3000 or acquired from 1.5T MRI scanners were not included in the study.

The T1‐weighted anatomical images were automatically processed using the FreeSurfer software version 6.0 (https://surfer.nmr.mgh.harvard.edu/).^22^ This provided outputs containing estimates of cortical volume, hippocampal volume, cortical thickness, white matter volume, and white matter hypointensities volume. Left and right hippocampal volumes were averaged. To account for head size differences, all volumes were expressed as a fraction of the total intracranial volume, namely cortical volume fraction (CVF), bilateral hippocampal volume fraction (HVF), white matter fraction, and white matter hypointensities fraction.

A cortical thickness (CT) “AD signature” previously determined using independent data from the Alzheimer's Disease Neuroimaging Initiative (ADNI)^23^ was calculated combining bilateral cortical regions: superior temporal, parahippocampal, middle temporal, inferior parietal, inferior temporal, fusiform, and precuneus.

DTI data were processed using the FMRIB software library (FSL Version 6.0.1, FMRIB, Oxford, UK, http://www.fmrib.ox.ac.uk/fsl/).^24^ Data were corrected for eddy current distortions and head motion,^25^ and the diffusion tensor model at each voxel was fitted using DTIFIT. To control for the effect of head motion in DTI maps, a displacement index was calculated using an in‐house script.

### 2.3 Cortical diffusivity analysis

Cortical diffusivity analysis was conducted using a proprietary software tool (Cortical Disarray Measurement [CDM]; patent WO2016162682A1). The software generates cortical profiles, that is, lines across the cortex in a radial direction, replicating columnar organization within the cortex.^12^, ^13^ Briefly, the metrics calculated were three measures relating to the components of diffusion: AngleR is the angle between the radial minicolumn axis and the principal diffusion direction (in radians), ParlPD is the principal diffusion component parallel with the radial minicolumns (× 10^−3^ mm^2^/s), and PerpPD^+^ combines the components perpendicular to the radial minicolumns (× 10^−3^ mm^2^/s). PerpPD^+^ used here is a variant of the earlier PerpPD used in Torso et al.^5^, ^13^ PerpPD^+^ includes multiple components (secondary and tertiary) orthogonal to the cortical columnar profile. All the cortical values were averaged to reduce the influence of noise in the DTI scans, effectively smoothing the data, and ensuring only directionality with some local coherence would dominate, guarding against the influence of random deflections from the radial direction. Values for the diffusion tensor–derived metrics were averaged along the cortical profiles, and then across cortical regions using the Desikan–Killiany cortical parcellation.

Values for the three cortical diffusivity metrics were averaged over bilaterally symmetric meta‐regions or “AD signatures” comprising medial–temporal and connected regions, previously determined using independent data from ADNI.^23^ This AD signature overlaps extensively with the CT AD signature described above but involves more medial posterior regions (e.g., posterior–cingulate).

### 2.4 Statistical analyses

Statistical analyses were performed using SPSS software (version 29.0; SPSS Inc.) and MATLAB (version 9.9.0; MathWorks, www.mathworks.com). Demographic and clinical values were investigated using chi‐square tests for categorical measures and analysis of variance tests for continuous values.

The CSF values available for a subset of participants, adjusted for age, sex, and assay methodology, were analyzed to explore potential associations with MRI metrics (cortical diffusivity whole brain and signatures, HVF, CT signature, and CVF using Pearson correlation, with corrections for multiple comparisons applied using the Benjamini–Hochberg false discovery rate (FDR).^26^ Given the small number of MCI participants (MCI NC = 8, MCI C = 15) with CSF values, the participants were pooled (CU, MCI, and AD).

Univariate generalized linear model (GLM) analyses were used to investigate differences among diagnostic groups in cortical diffusion measurements at whole‐brain and signature level, using the diagnostic group as a fixed factor, adjusting for scanner model, b‐value, age, sex. The MRI macrostructural measures were assessed using the same model, excluding the adjustment for b‐value. Reported significant results were adjusted for multiple comparisons using the Bonferroni correction.

Regional differences were investigated using linear models, with the cortical diffusivity measures (AngleR, PerpPD^+^, ParlPD) as dependent variables; clinical diagnosis as fixed factor; and controlling for scanner model, b‐value, age, sex. The results reported were corrected for multiple comparisons to control FDR over the set of Desikan–Killiany regions.

To better focus the comparison between different MRI measures in the survival analysis, only the AD signature of the cortical diffusivity measure that best distinguishes the two MCI groups at the regional level will be included. Regarding macrostructural measures, those included in the analysis represent the most studied and commonly used for exploring hippocampal damage (HVF), the cortical AD signature (CT signature), and global diffuse atrophy (CVF).

Although various micro‐ and macrostructural measures could be considered in the study, the measures included in the comparison aim to account for the spatial progression of AD‐type neurodegenerative processes, starting from localized hippocampal damage, progressing to temporal lobe involvement, and ultimately leading to global cortical involvement.

For survival analysis, each value of the compared measures (PerpPD^+^ signature, HVF, CT signature, and CVF) were adjusted for confounds (age, sex, scanner model, and b‐value) using linear models and then dichotomized into neurodegeneration positive (N^+^) and negative (N^−^) values using a cutpoint determined as the crossing point of the estimated probability density functions of the CU and AD groups.^27^ This intersection point represents the threshold that minimizes classification errors between the two groups. All PerpPD^+^ signature values equal or greater than this value were considered neurodegeneration positive (N^+^) while HVF, CT signature, and CVF values below the cutpoint were considered neurodegeneration positive (N^+^).

Survival functions were generated to estimate the probability of remaining in the MCI state or progressing to dementia over time. Progression‐free survival was visualized using Kaplan–Meier survival curves, with separate curves for the dichotomized MCI subgroups (N^+^ and N^−^), using cortical diffusivity or macrostructural measures.

Median survival time and survival probabilities at specific time points were reported.

Differences in survival curves between groups were formally tested using the log‐rank test. Statistical significance was set at *P* < 0.05.

To compare the predictive ability of the MRI measures considered (PerpPD^+^ signature, HVF, CT signature, and CVF) the pairwise log‐rank test was used.

## 3 RESULTS

### 3.1 Participant characteristics

Clinical and demographic characteristics of the studied cohort are summarized in Table 1. All patient groups are significantly different from the CU group, for age and sex distribution. The distribution of *APOE* ε4 carriers and non‐carriers differed significantly between the groups, with a higher proportion of *APOE* ε4 carriers observed in the AD and MCI C group. Significant differences were observed between the groups in MMSE, global CDR, and CDR‐SB scores, with the AD group exhibiting a higher level of impairment as expected. The MCI NC group showed a significantly higher proportion of Hispanic individuals.

Pearson correlation analysis revealed multiple significant associations of CSF biomarkers with structural MRI. The results are summarized in Table 2.

**TABLE 2:** Pearson correlation results between CSF biomarkers and MRI metrics.

CSF/MRI correlations			
	Aβ1–42	P‐tau181	T‐tau
Cortical Volume Fraction	*r* = 0.311, *P* < 0.001^*^	*r* = 0.020, *P* = n.s.	*r* = 0.036, *P* = n.s.
Cortical Thickness Signature	*r* = 0.295, *P* < 0.001^*^	*r* = –0.227, *P* = 0.003^*^	*r* = –0.210, *P* = 0.007^*^
Hippocampal Volume Fraction	*r* = 0.247, *P* = 0.001^*^	*r* = –0.191, *P* = 0.014^*^	*r* = –0.177, *P* = 0.023^*^
AngleR Whole‐brain	*r* = –0.260, *P* < 0.001^*^	*r *= 0.190, *P* = 0.014^*^	*r* = 0.244, *P* = 0.002^*^
PerpPD^+^ Whole‐brain	*r* = –0.196, *P* = 0.012^*^	*r* = 0.247, *P* < 0.001^*^	*r* = 0.231, *P* = 0.003^*^
ParlPD Whole‐brain	*r* = –0.079, *P* = n.s	*r* = 0.074, *P* = n.s.	*r* = 0.044, *P* = n.s.
AngleR Signature	*r* = –0.404, *P* < 0.001^*^	*r* = 0.185, *P* = 0.017^*^	*r* = 0.245, *P* = 0.001^*^
PerpPD^+^ Signature	*r* = –0.289, *P* < 0.001^*^	*r *= 0.277, *P* < 0.001^*^	*r* = 0.339, *P* < 0.001^*^
ParlPD Signature	*r* = –0.118, *P* = n.s.	*r *= 0.050, *P* = n.s.	*r *= 0.030, *P* = n.s.

*Note*: This table presents the Pearson correlation coefficients between CSF biomarkers (including amyloid, total tau, and phosphorylated tau) and micro and macrostructural MRI metrics.

Abbreviations: Aβ, amyloid beta; CSF, cerebrospinal fluid; FDR, false discovery rate; MRI, magnetic resonance imaging; p‐tau, phosphorylated tau; t‐tau, total tau.

* Statistically significant after Benjamini–Hochberg FDR correction.

### 3.2 Cortical diffusivity analysis: whole‐brain and signature

Table 3 summarizes whole‐brain and signature microstructural data. At the whole‐brain level, the GLM revealed higher AngleR, PerpPD^+^, and ParlPD values in the AD group than in the CU group. PerpPD^+^ and ParlPD values were able to differentiate significantly between AD and the other two MCI groups, and the MCI NC and CU groups. AngleR and PerpPD^+^ were able to differentiate significantly between the MCI C and the CU group.

**TABLE 3:** Whole‐brain and microstructural signature.

	CU *n* = 474	MCI NC *n* = 110	MCI C *n* = 86	AD *n* = 156
**Microstructural whole‐brain**				
AngleR	0.884 (0.4)	0.894 (0.4)	0.918 (0.4)^*^	0.922 (0.5)^*^, ^†^
PerpPD^+^	2.081 (0.19)	2.135 (0.30)^*^	2.241 (0.22)^*^, ^†^	2.240 (0.33)^*^, ^†^, ^‡^
ParlPD	0.581 (0.4)	0.597 (0.7)^*^	0.602 (0.6)^†^	0.601 (0.7)^*^, ^†^, ^‡^
**Microstructural Signature**				
AngleR	0.935 (0.04)	0.949 (0.04)	1.001 (0.05)^*^, ^†^	1.014 (0.06)^*^, ^†^
PerpPD^+^	1.986 (0.17)	2.044 (0.27)	2.172 (0.22)^*^, ^†^	2.179 (0.30)^*^, ^†^, ^‡^
ParlPD	0.619 (0.05)	0.639 (0.08)^*^	0.649 (0.06)^†^	0.648 (0.08)^*^, ^†^, ^‡^

* Significantly different compared to control (Bonferroni‐corrected *P*  <  0.05).

^†^ Significantly different compared to MCI NC (Bonferroni‐corrected *P*  <  0.05).

^‡^ Significantly different compared to MCI C (Bonferroni‐corrected *P*  <  0.05).

Abbreviations: AD, Alzheimer's disease; CU, cognitively unimpaired; MCI C, mild cognitive impairment converter; MCI NC, mild cognitive impairment non‐converter.

Similarly, the signature results revealed that AngleR, PerpPD^+^, and ParlPD values were significantly higher in the AD group than in CU group. PerpPD^+^ and ParlPD values were able to differentiate significantly between the AD group and the two MCI groups, while all the signature measures were able to differentiate between MCI NC and MCI C. The GLM results also revealed that AngleR and PerpPD^+^ were able to differentiate significantly between the MCI C and the CU group, while only ParlPD was able to differentiate the MCI NC and CU groups.

### 3.3 Cortical diffusivity regional analysis

After FDR correction, regional results show a progressive increase in AngleR values along the AD continuum (Figure S1 in supporting information). Comparing each patient group to the CU group, the results demonstrate a pattern of significant differences primarily in temporal regions (such as the superior temporal area) in the comparison between MCI NC and CU. A progressive expansion of the cortical alteration pattern is observed, predominantly affecting temporal, frontal, and parietal regions in MCI C patients. This extensive pattern of cortical alteration reaches its peak in the comparison between CU and AD, with significant differences involving almost all cortical regions. In the comparison between the two MCI groups, the results show a significant increase in AngleR values in key regions, such as the bilateral precuneus, in the MCI converter group. Finally, comparing the MCI converter group to the AD group, the results show a predominantly temporal pattern of significantly increased AngleR values, particularly bilaterally in the superior temporal regions, middle temporal regions, isthmus cingulate, and posterior cingulate regions.

**FIGURE 1:**
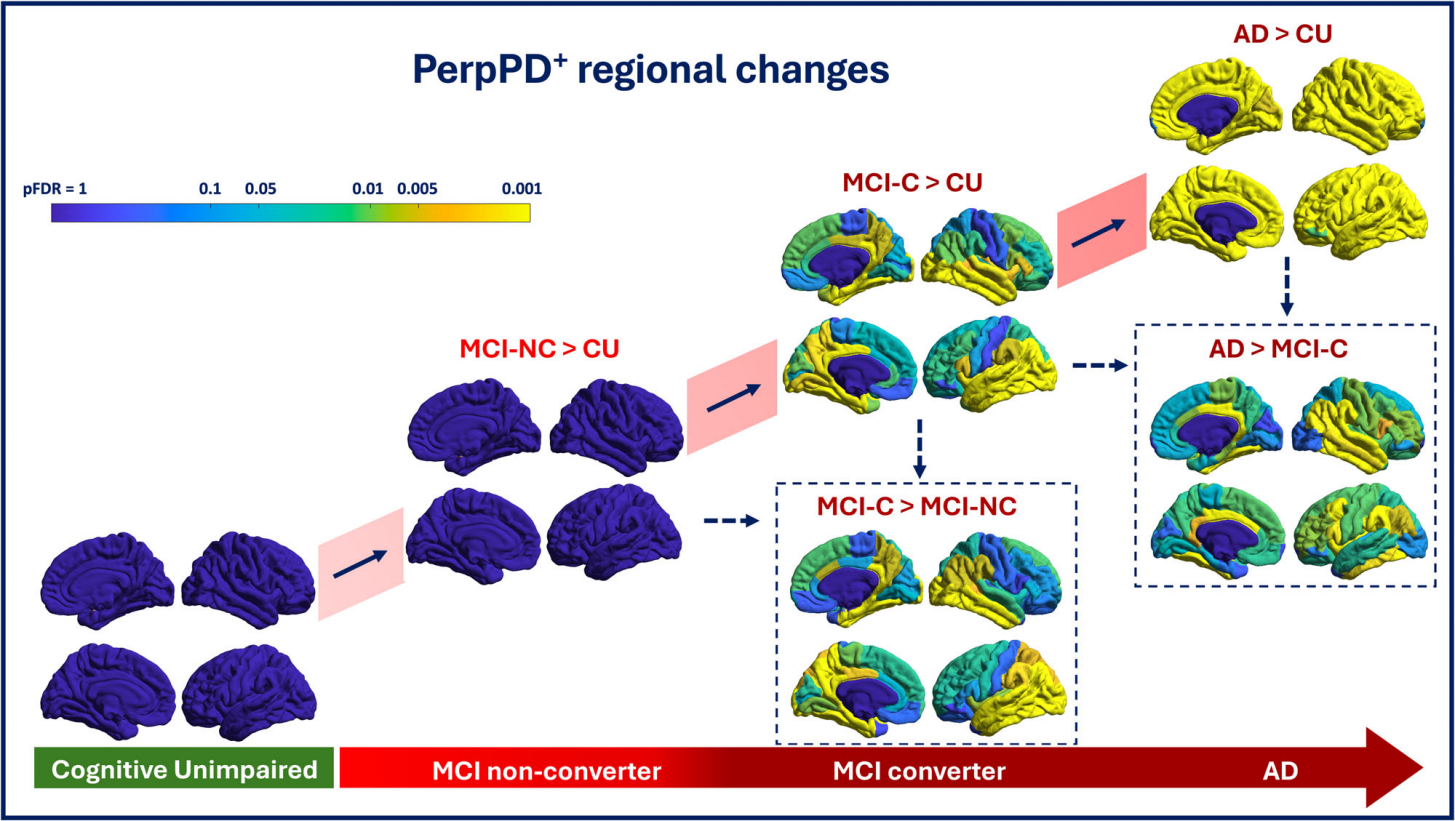
Cortical regions exhibiting significant increases in PerpPD^+^ values. The regions are highlighted on a standardized brain template, with color intensity indicating the magnitude of the statistical significance corrected for multiple comparisons using the false discovery rate (FDR). AD, Alzheimer's disease; CU, cognitively unimpaired; MCI C, mild cognitive impairment converter; MCI NC, mild cognitive impairment non‐converter

Regarding PerpPD^+^ values, regional results show a progressive and significant increase in PerpPD^+^ values in the MCI C and AD groups (Figure 1). Compared to the CU group, the MCI C group exhibits a broad bilateral pattern of elevated PerpPD^+^ values, primarily affecting the bilateral temporal regions, followed by elevated values in parietal and frontal areas. In the AD group, the results indicate a significant increase in PerpPD^+^ values across all cortical regions. Comparing the two MCI groups, the regional analysis reveals a significant increase in PerpPD^+^ values in the MCI C group, mainly in bilateral temporal areas, with additional involvement of parietal and frontal regions. A broad temporo‐parieto‐frontal pattern of increased PerpPD^+^ values is also found in the AD group compared to the MCI C group.

Finally, the regional analysis of ParlPD values (Figure S2 in supporting information) reveals a significant increase in the MCI C and AD groups, predominantly in inferior temporal and occipital areas. The same pattern is observed comparing the two MCI groups, while no significant differences are found comparing the MCI C group to the AD group. At the same time, the results highlight a significant progressive reduction in ParlPD values (Figure S3 in supporting information) along the clinical continuum. The areas showing reduced ParlPD values are the insula and superior temporal regions bilaterally.

### 3.4 Survival analysis

The positivity for PerpPD^+^ signature, HVF, CT signature, and CVF values, determined in CU and AD groups using the cut‐off corresponding at the crossing point of the distribution of the two groups, was applied to classify MCI participants in N^−^ and N^+^.

**FIGURE 2 alz70310-fig-0002:**
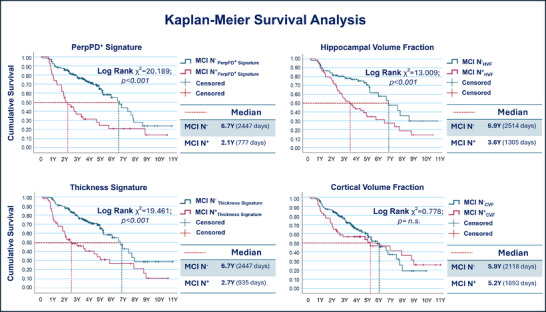
Survival curves for each measure, where each point represents the proportion of MCI participants that have not yet progressed to dementia at different time points (years). CVF, cortical volume fraction; HVF, hippocampal volume fraction; MCI, mild cognitive impairment

Kaplan–Meier estimates (Table 4) using PerpPD^+^ signature values revealed a significantly lower survival time (median 777 days, 95% confidence interval [CI]: 503.8–1050.2) for N^+^ compared to N^−^ (median 2447 days, 95% CI: 2052.3–2841.7). Kaplan–Meier survival curves were plotted (Figure 2), and the log‐rank test showed significant differences in clinical progression over time between the two MCI groups (chi‐square = 20.189; *P* value < 0.001).

**TABLE 4:** Kaplan–Meier estimates.

	Mean (days)^a^				Median (days)			
			95% confidence interval				95% confidence interval	
	Estimate	Std. error	Lower bound	Upper bound	Estimate	Std. error	Lower bound	Upper bound
**N^‐^_PerpPD_+ _signature_**	*2356.1*	153.8	2054.7	2657.5	*2447.0*	201.3	2052.3	2841.7
**N^+^_PerpPD_+ _signature_**	*1414.6*	209.1	1004.8	1824.4	*777.0*	139.4	503.8	1050.2
**N^‐^_HVF_**	*2490.1*	186.1	2126.1	2032.6	*2514.0*	334.5	1858.4	3169.5
**N^+^_HVF_**	*1710.5*	164.6	1388.3	2419.9	*1305.0*	167.3	939.1	1670.9
**N^‐^_Thickness Signature_**	*2415.9*	167.5	2087.6	2744.2	*2447.0*	195.3	2064.2	2829.8
**N^+^_Thickness Signature_**	*1518.8*	193.4	1139.7	1897.9	*935.0*	285.7	375.1	1494.9
**N^‐^_CVF_**	*2053.5*	133.4	1792.1	2314.9	*2118.0*	231.7	1663.8	2572.2
**N^+^_CVF_**	*2088.4*	225.4	1646.7	2530.2	*1893.0*	736.1	450.4	3335.6

Abbreviations: CVF, cortical volume fraction; HVF, hippocampal volume fraction.

^a^ Estimation is limited to the largest survival time if it is censored.

Concerning HVF, the analysis revealed a significantly lower survival time (median 1305 days, 95% CI: 939.1–1670.9) for MCI N^+^ compared to N^−^ (median 2514 days, 95% CI: 1858.4–3169.5). The log‐rank test showed significant differences in clinical progression over time between the two MCI groups (chi‐square = 13.009; *P* value < 0.001).

For the CT signature, the MCI N^+^ group showed a significantly lower survival time (median 935 days, 95% CI: 375.1–1494.9) compared to N^−^ (median 2447 days, 95% CI: 2064.2–2829.8). The log‐rank test showed significant differences in clinical progression over time between the two MCI groups (chi‐square = 19.461; *P* value < 0.001).

Finally, regarding the CVF, the analysis revealed no significant difference in the survival time between the MCI N^+^ (median 1893 days, 95% CI: 450.4–3335.6) and the N^−^ groups (median 2118 days, 95% CI: 1663.8–2572.2). The log‐rank test showed a non‐significant difference in clinical progression over time between the two MCI groups (chi‐square = 0.778; *P* value > 0.05).[Fig alz70310-fig-0002]

The cumulative proportion surviving (CPS) at the time was calculated to evaluate the survival function over time for PerpPD^+^ signature, HVF, CT signature, and CVF and summarized in the Table 5

**TABLE 5 alz70310-tbl-0005:** Cumulative proportion of MCI participants that have not yet progressed to dementia.

PerpPD^+^ Signature										
	**0**	**1Y**	**2Y**	**3Y**	**4Y**	**5Y**	**6Y**	**7Y**	**8Y**	**9Y**
**Cumulative proportion surviving at the time**										
Negative	1	0.93	0.86	0.79	0.72	0.65	0.55	0.44	0.28	0.24
Positive	1	0.79	0.55	0.37	0.31	0.24	0.21	0.21	0.21	0.14
**Number of participants available at each time point**										
Negative	154	140	127	91	64	38	17	11	7	3
Positive	42	33	23	14	10	7	6	4	3	1

Hippocampal Volume Fraction										
	**0**	**1Y**	**2Y**	**3Y**	**4Y**	**5Y**	**6Y**	**7Y**	**8Y**	**9Y**
**Cumulative proportion surviving at the time**										
Negative	1	0.91	0.81	0.79	0.75	0.71	0.58	0.48	0.36	0.30
Positive	1	0.89	0.76	0.55	0.44	0.35	0.31	0.27	0.19	0.14
**Number of participants available at each time point**										
Negative	122	108	94	69	48	28	15	8	6	3
Positive	74	65	55	36	22	15	8	6	4	2

**Cortical Thickness Signature**										
	**0**	**1Y**	**2Y**	**3Y**	**4Y**	**5Y**	**6Y**	**7Y**	**8Y**	**9Y**
**Cumulative proportion surviving at the time**										
Negative	1	0.96	0.88	0.79	0.72	0.65	0.55	0.43	0.29	0.29
Positive	1	0.75	0.59	0.46	0.40	0.31	0.27	0.27	0.21	0.11
**Number of participants available at each time point**										
Negative	140	132	118	86	59	33	17	10	6	6
Positive	56	42	32	19	14	10	6	6	4	2

Cortical Volume Fraction										
	**0**	**1Y**	**2Y**	**3Y**	**4Y**	**5Y**	**6Y**	**7Y**	**8Y**	**9Y**
**Cumulative proportion surviving at the time**										
Negative	1	0.93	0.85	0.76	0.65	0.56	0.45	0.37	0.19	0.19
Positive	1	0.84	0.66	0.57	0.57	0.50	0.46	0.41	0.36	0.26
**Number of participants available at each time point**										
Negative	139	126	113	78	48	29	13	9	3	3
Positive	57	48	37	28	25	15	9	8	7	5

**FIGURE 3 alz70310-fig-0003:**
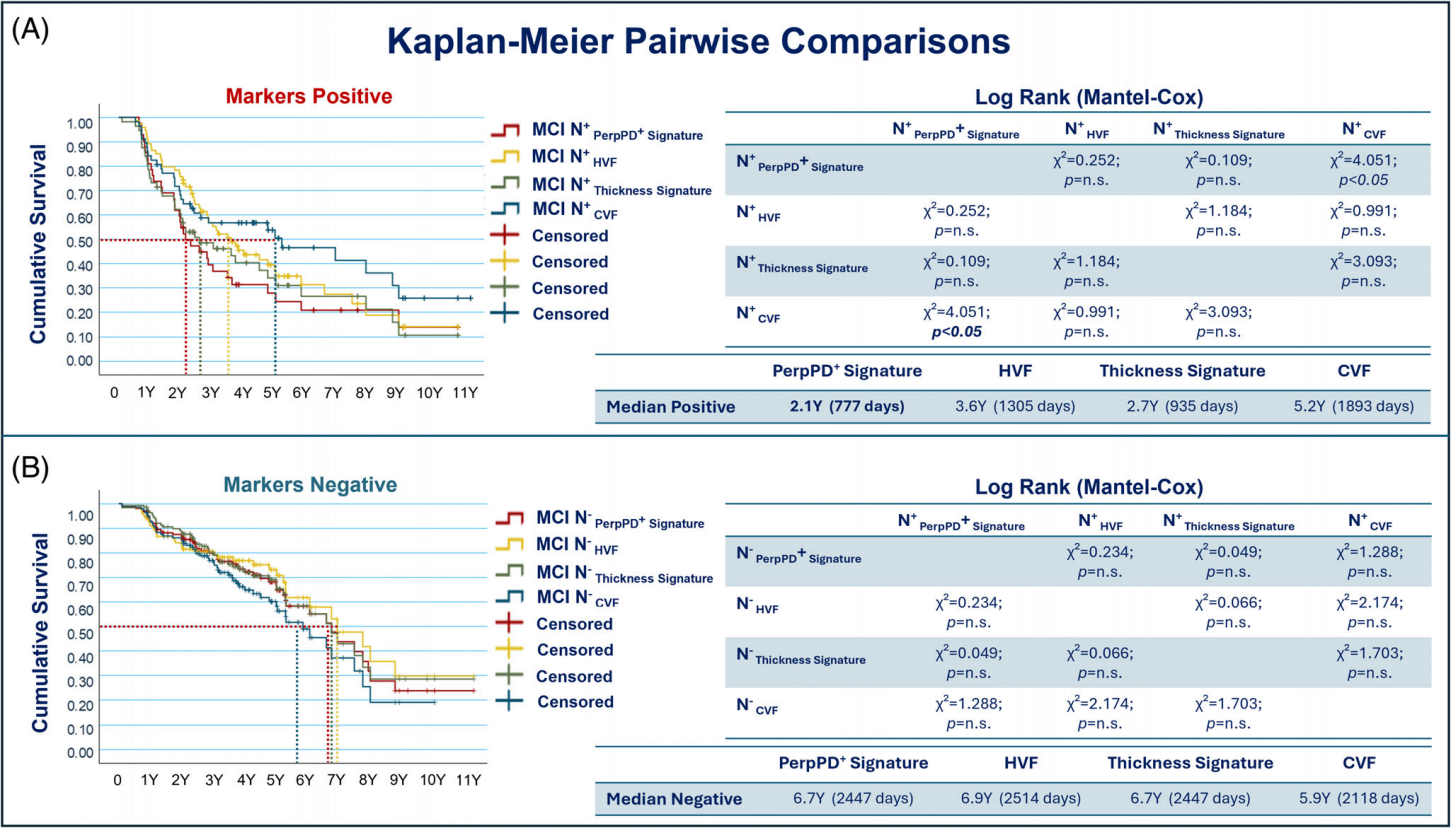
A, Comparison of survival curves for subjects classified as positive according to each of the four measures being compared. B, Comparison of survival curves for subjects classified as negative. The tables report the chi‐square values and *P* values for each pairwise comparison between the survival curves. CVF, cortical volume fraction; HVF, hippocampal volume fraction; MCI, mild cognitive impairment

Two separate Kaplan–Meier pairwise comparison analyses were performed to assess significant differences between survival curves for each of the four measures compared (Figure 3). One analysis compared the positive curves of each measure, while the other focused on the negative curves. This approach allowed for a detailed evaluation of whether survival distributions differed significantly across markers within each subgroup.

The log rank (Mantel–Cox) analysis performed to compare the N^+^ curves showed that only the PerpPD^+^ signature curve was significantly different compared to cortical volume fraction (chi‐square = 4.051; *P* value *** < *** 0.05). No other significant difference was found in the comparison between curves.

## 4 DISCUSSION

Our main goal was to investigate the ability of cortical diffusivity measures to detect microstructural alterations along the clinical continuum from CU to dementia and to assess their potential for predicting progression from MCI to dementia using survival analysis.[Fig alz70310-fig-0003], [Table alz70310-tbl-0005]

Previous studies have shown that amyloid and particularly tau PET are promising biomarkers for predicting progression to dementia.^28^, ^29^ In this study we investigate the utility of microstructural and macrostructural neurodegeneration biomarkers.

Brain imaging biomarkers are highly useful for detecting neurodegenerative processes.^2^, ^30^ However, the most widely used MRI biomarkers rely on macrostructural measures, which, while robust in differentiating healthy individuals from patients in more advanced disease stages, are less accurate in predicting disease progression or differentiating groups in the earlier stages.^31^ It is understood^32^ that AD pathology can lead to alterations in cortical architecture at the microstructural level before clearly detectable changes in macrostructural measures like volume.

Hippocampal volume, often expressed as a fraction of brain or intracranial volume, is commonly used in research, trials, and clinical practice.^33^ However, while hippocampal volume measurement is informative about the presence (and/or coexistence) of AD,^33^, ^34^ and is a biomarker for monitoring longitudinal change,^33^ it has limitations regarding the progression from MCI to AD,^35^ due to substantial variation in hippocampal volume and its reduction across MCI patients. Based on the staging proposed by Braak and Braak,^36^, ^37^ it is apparent that hippocampal abnormality, along with alterations in adjacent cortical areas (e.g., entorhinal, parahippocampal), represents one of the first manifestations of AD progression. However, for a better prediction of disease evolution, it is crucial to go beyond the hippocampal region and consider additional areas included in Braak staging, while still focusing on the temporal lobe.^38^ For this reason, in the present study, we investigated also the CT signature and the CVF.

Recent studies^5^, ^8^, ^20^ have shown that microstructural imaging measures offer the possibility of identifying neurodegenerative alterations earlier than macrostructural measures. However, these studies have not investigated time of progression from MCI to dementia using survival analysis, which motivated the present study.

### 4.1 Patterns of cortical alteration across clinical stages

In the first part of the study, we explored microstructural alterations at the whole brain, macroregional (represented by an AD signature), and regional levels in patients along the clinical continuum from CU individuals through MCI non‐converters, MCI converters, to AD dementia. We tested whether diffusion measures can detect progressive cortical alterations and identified the measure potentially most useful for predicting the progression from MCI to dementia.

Regional results show how AngleR can detect the progressive microstructural changes at each clinical stage. Specifically, AngleR results demonstrate that the initial fronto‐temporal pattern of microstructural differences observed in the comparison between MCI NC and CU groups gradually expands to involve the entire cortex in dementia patients compared to CU participants. PerpPD^+^ results also reveal a progressive broadening of microstructural cortical alterations, eventually affecting the entire cortex in dementia patients. However, the comparison of PerpPD^+^ values between MCI NC and CU groups does not show the significant differences detected by AngleR. This may be due to the different sensitivity of the two measures to cortical alterations caused by distinct degenerative proteins. A previous study with a cohort of autopsy‐confirmed patients^11^ demonstrated that AngleR values are associated with amyloid deposition (measured as Thal phase), while PerpPD^+^ values are associated with tau deposition (measured as Braak stage). From this, it can be inferred that AngleR may be sensitive to early alterations driven by amyloid plaques, while PerpPD^+^ may be more sensitive to the disruption of minicolumns caused by neuronal pathology due to tau deposition, generally associated with more pronounced symptomatology. In line with this, PerpPD^+^ shows greater capacity in differentiating MCI NC and MCI C groups, with the regional pattern demonstrating more sensitive detection of the microstructural damage underlying worsening symptoms and the consequent clinical progression (conversion to dementia).

ParlPD values also show a progressive increase along the clinical continuum in inferior temporal, occipital, and frontal regions. However, the results reveal an intriguing progressive pattern of ParlPD value reduction in the insular cortex and the superior temporal gyrus bilaterally. This distinct pattern of reduced ParlPD values in these regions may reflect a combination of factors related to their structure, function, and vulnerability to neurodegenerative pathologies. Areas with predominantly granular cortical layers,^39^, ^40^ such as the superior temporal gyrus, are characterized by more integrated minicolumns than regions with dysgranular cortical layers, such as the insula. Furthermore, being regions involved in the processing of complex information, they are characterized by neurons with extensive dendritic arborizations. This makes them vulnerable to the loss of long‐range connectivity during AD, in which the degeneration of associative pathways is an early hallmark.^41^, ^42^ Previous studies have shown that ParlPD is sensitive to damage in white matter tracts,^11^, ^12^ and this reduction might reflect a progressive deafferentation of the cortical regions.

Taken together, PerpPD^+^ emerged as the measure most useful for investigating conversion to AD within the MCI group. More specifically, a PerpPD^+^ signature was used, representing a predefined meta‐region that includes cortical areas considered key in the progression of AD.^25^

### 4.2 Risk prediction

The Kaplan–Meier survival analysis revealed that PerpPD^+^ signature was a superior predictor of conversion to dementia compared to other structural measures, such as HVF, CT signature, and CVF. Participants who were positive for PerpPD^+^ signature exhibited a median survival time of ≈ 1.5 years earlier than those identified through reduced hippocampal volume, 0.6 years earlier than those identified through reduced CT signature, and 3.1 years earlier than those identified through reduced CVF, while the four measures had similar median survival times in their neurodegeneration‐negative groups.

The performance advantage of PerpPD^+^ signature may be attributed to its ability to capture a broader spectrum of AD‐related changes in neocortical regions beyond the hippocampus, in line with prior evidence^11^ indicating that increasing PerpPD^+^ detects the widespread cortical damage across increasing Braak stages. In contrast, hippocampal volume, while a robust marker for MCI and AD, may primarily reflect localized damage and is likely more sensitive to the presence—rather than progression—of AD pathology.

Regarding the CT signature and CVF, previous studies^5^ have shown that cortical microstructural measures can predict macrostructural cortical changes. This finding may explain the superior performance of the PerpPD^+^ signature in predicting the progression from MCI to dementia.

From a clinical perspective, the earlier detection of AD risk using PerpPD^+^ signature could inform timely interventions and improve the design of therapeutic trials aimed at slowing disease progression. As shown by the Kaplan–Meier curves, patients with a clinical diagnosis of MCI who are PerpPD^+^ positive and negative exhibit a significant difference in conversion risk even within the first years of follow‐up (median conversion for MCI+ at 2.1 years vs. 6.7 years for MCI–). Depending on the characteristics and objectives of a potential pharmacological treatment, PerpPD^+^ positivity or negativity could serve as a valuable marker for stratifying and selecting patients most suitable for a specific type of intervention. For example, PerpPD^+^ positive patients, who are at high risk of conversion within the next 2 years, might or might not be ideal candidates for a disease‐modifying treatment (DMT) targeting the earliest stages of neurodegeneration (e.g., anti‐amyloid treatments). These patients could represent the most appropriate population for treatments with a more symptomatic focus or for DMTs with targets more closely coupled to symptoms (e.g., anti‐tau treatments or treatments that aim to directly slow aspects of neurodegeneration). Conversely, PerpPD^+^ negative patients might be the most promising responders to DMTs focused on the earliest stages of a pathological cascade.

It is also important to consider the utility of a multimodal diagnostic approach to AD, in which combining biomarkers, such as amyloid and tau biomarkers that reflect distinct aspects of disease pathology, could enhance predictive accuracy, and/or could allow more granular stratification. In a real‐world memory clinic setting,^20^ microstructural biomarkers of neurodegeneration proved prognostically useful for MCI cases who were negative for amyloid and tau biomarkers.

While these findings offer important insights, there are limitations to consider. The survival curve analysis was based on the classification of participants into biomarker‐positive and biomarker‐negative groups, which might oversimplify the continuous nature of these measures. The small number of cases with amyloid and tau biomarkers available precluded consideration of the combination of these biomarkers with the microstructural and macrostructural neurodegeneration biomarkers considered here. Furthermore, while PerpPD^+^ signature outperformed other macrostructural measures in this study, the heterogeneity of the MCI population and differences in cortical regional vulnerability warrant further exploration. For example, future studies might further evaluate region‐specific alteration patterns or combine cortical diffusivity measures with other biomarkers such as amyloid or tau PET to refine predictions.

### 4.3 Conclusion

In conclusion, this study demonstrates the value of cortical diffusivity as a biomarker for predicting conversion from MCI to dementia. Its shorter median survival and superior performance compared to macrostructural MRI measures emphasize the need to incorporate cortical diffusivity assessments into clinical and research frameworks aimed at understanding and managing AD progression. Further research is needed to confirm these results in diverse cohorts and to explore the combination of multiple biomarkers.

## CONFLICT OF INTEREST STATEMENT

SAC is a co‐founder of a company, Oxford Brain Diagnostics, from which he has received funding for the research and preparation of this manuscript. MT, PK, and GR are currently employed at a company, Oxford Brain Diagnostics. SAC has a patent (WO2016162682A1) related to the diffusion MRI analysis used in the present study. Author disclosures are available in the supporting information.

## CONSENT STATEMENT

Informed written consent to take part in the study was collected from all participants or their caregivers at each respective ADRC.

## Supporting information



Supporting Information

Supporting Information
