# Supplementary material for: Predicting progression from MCI to dementia using cortical disarray measurement from diffusion MRI
Source: Alzheimers Dement. 2025 May 26;21(5):e70310. doi: 10.1002/alz.70310 (PMC12106053; doi:10.1002/alz.70310)
Supplement: Supplementary file 1 — Supporting Information [file ALZ-21-e70310-s002.docx]

**Supplementary Material**

**Figure 1. Cortical regions showing significant increases in AngleR values**


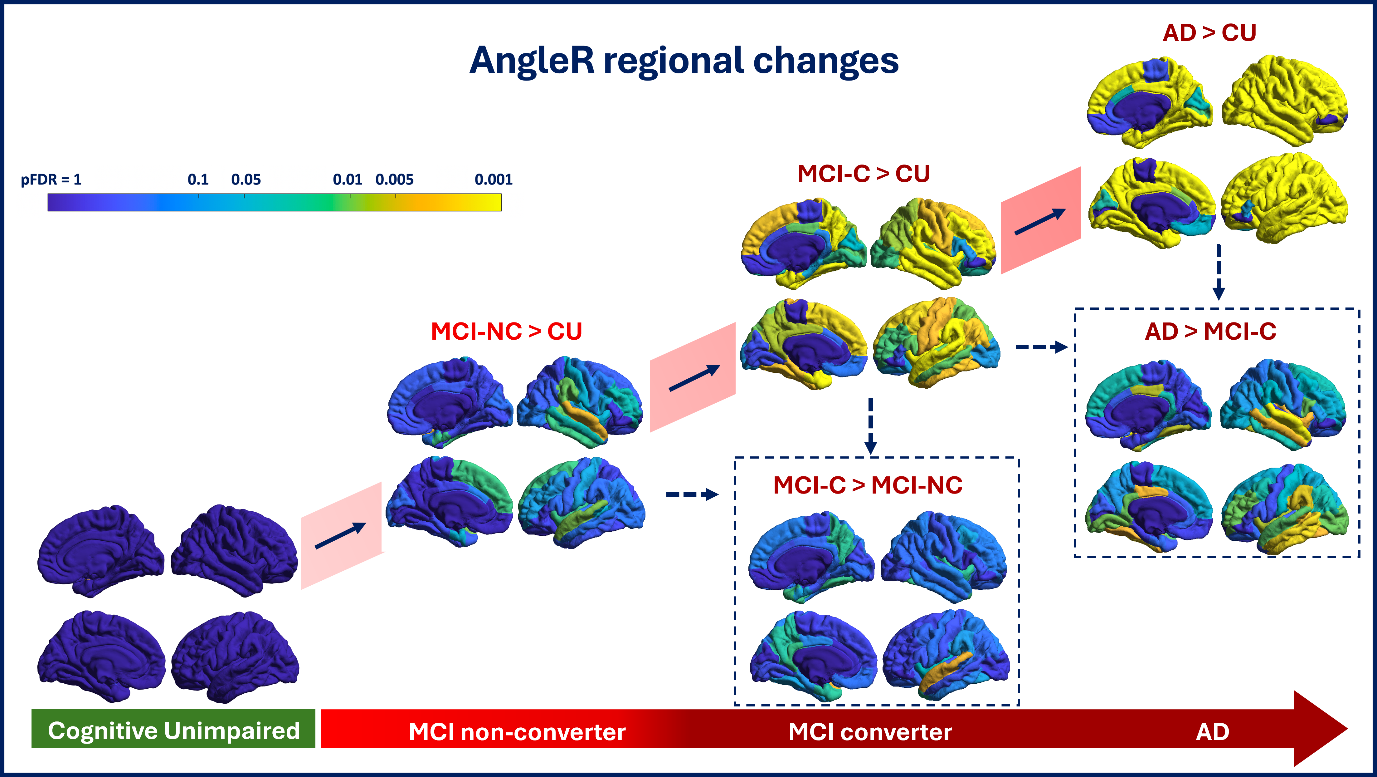


**Figure 1:** Cortical regions exhibiting significant increases in AngleR values. The regions are highlighted on a standardized brain template, with colour intensity indicating the magnitude of the statistical significance corrected for multiple comparisons using the false discovery rate (FDR).

**Figure 2. Cortical regions showing significant increases in ParlPD values**


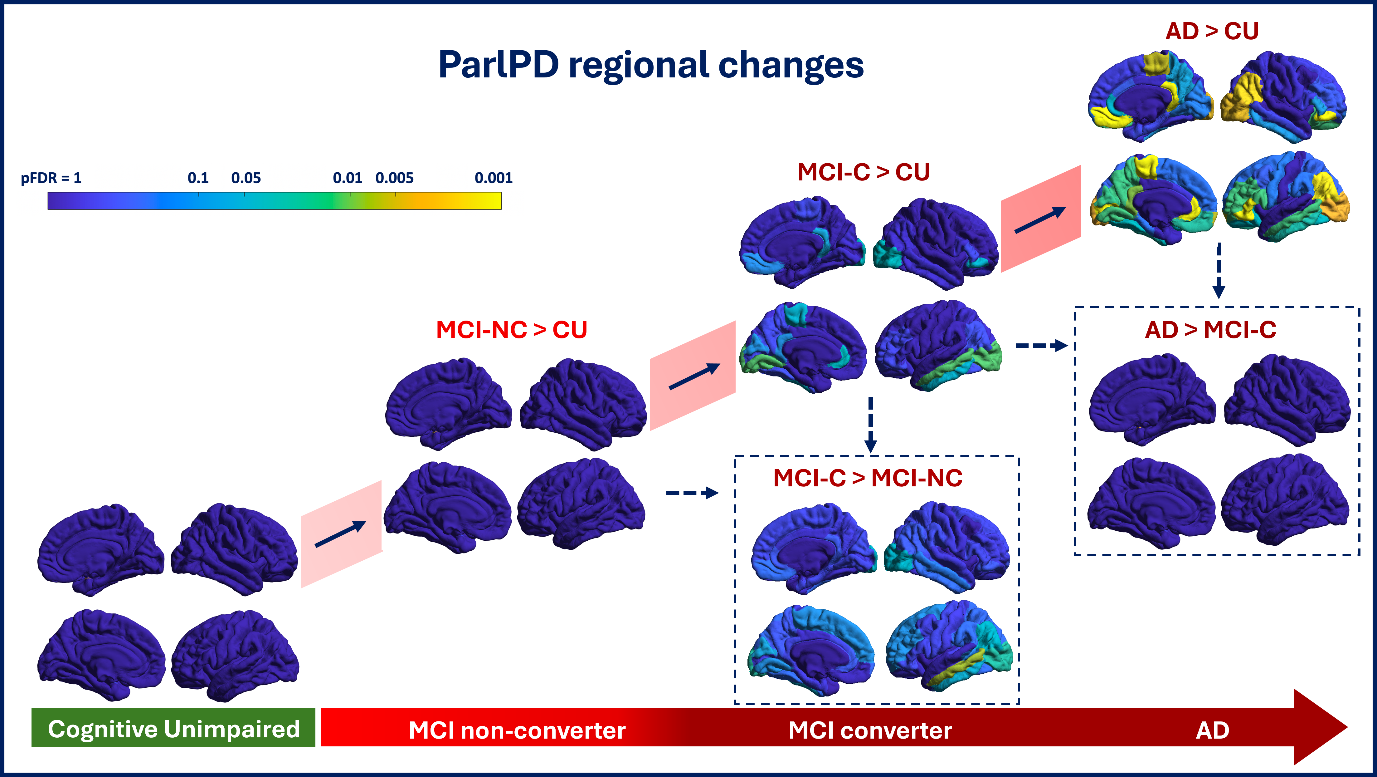


**Figure 2:** Cortical regions exhibiting significant increases in ParlPD values. The regions are highlighted on a standardized brain template, with colour intensity indicating the magnitude of the statistical significance corrected for multiple comparisons using the false discovery rate (FDR)

**Figure 3. Cortical regions showing significant decreases in ParlPD values**


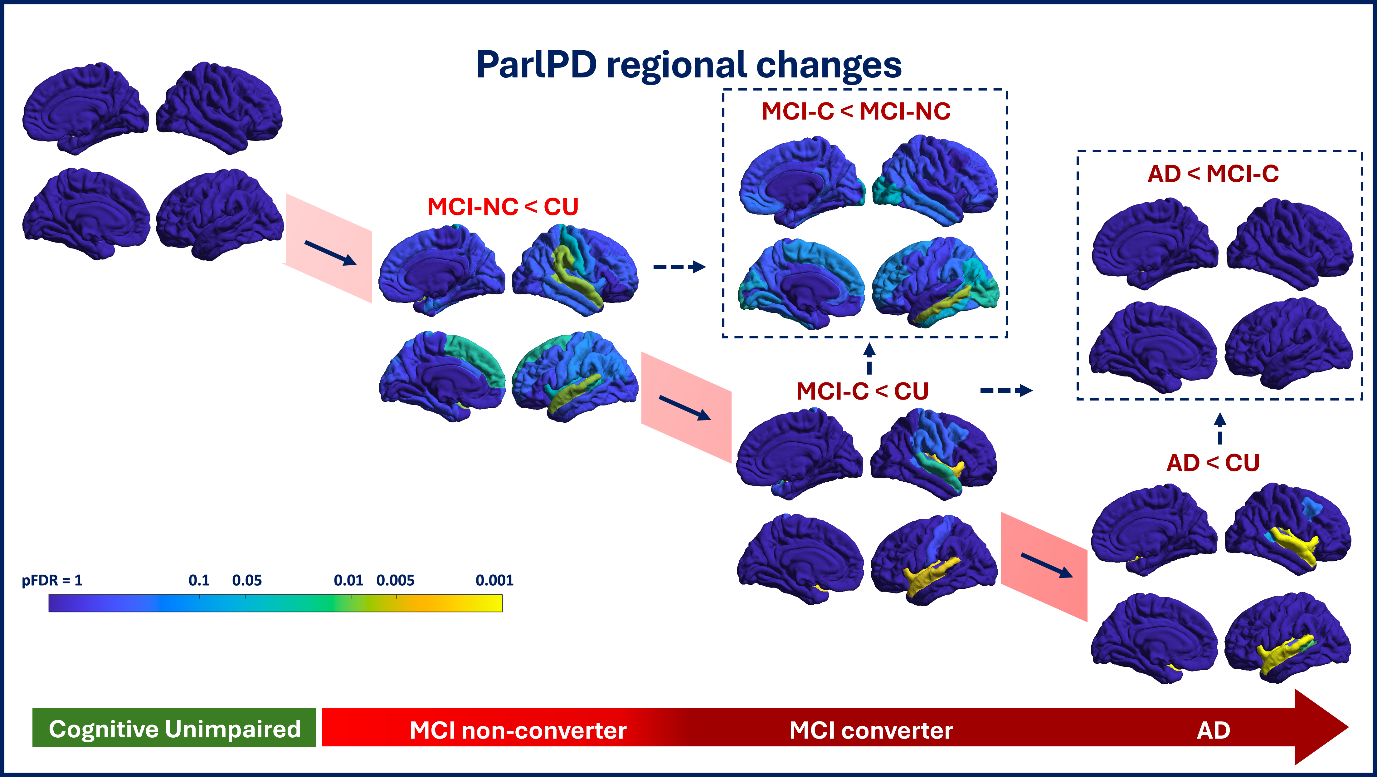


**Figure 3:** Cortical regions exhibiting significant decreases in ParlPD values. The regions are highlighted on a standardized brain template, with colour intensity indicating the magnitude of the statistical significance corrected for multiple comparisons using the false discovery rate (FDR).
